# Usefulness of Flow Cytometry Monocyte Partitioning in the Diagnosis of Chronic Myelomonocytic Leukemia in a Real-World Setting

**DOI:** 10.3390/cancers17071229

**Published:** 2025-04-05

**Authors:** Yijie Liu, Hamza Tariq, Lucy Fu, Juehua Gao, Taruna Jagtiani, Kristy Wolniak, Barina Aqil, Peng Ji, Yi-Hua Chen, Qing Ching Chen

**Affiliations:** 1 Department of Pathology, Northwestern University Feinberg School of Medicine & Northwestern Memorial Hospital, Chicago, IL 60611, USAqchen@nm.org (Q.C.C.); 2 Clinical Flow Cytometry Laboratory, Department of Pathology, Northwestern Memorial Hospital, Chicago, IL 60611, USA

**Keywords:** CMML, flow cytometry, monocytes partitioning

## Abstract

Background: Based on CD14/CD16 expression, monocytes can be divided into the following three functionally distinct subsets: classical (MO1, CD14++/CD16-), intermediate (MO2, CD14+/CD16+) and non-classical (MO3, CD14^dim^/CD16-). An expanded MO1 subset (cutoff, ≥94%) was found to be predictive of CMML. However, the utility of this test in routine practice has important limitations, with some reporting low sensitivity or a lack of correlation. Here, we sought to evaluate the practical usefulness of this test by using our routine antibody panel and a new gating strategy. Methods: Our study included 56 peripheral blood (PB) and 69 bone marrow (BM) samples. The PB cohort included 20 patients with CMML, 21 with no myeloid neoplasms (non-MN) and 15 with other myeloid neoplasms (non-CMML-MN). The BM cohort included 25 CMML, 16 non-MN and 28 non-CMML-MN cases. Taking advantage of an existing 8-color myelomonocytic tube routinely used in our lab, we conducted a retrospective monocyte subset analysis using a new sequential gating strategy. Results: The assay was able to distinguish CMML from non-CMML cases with high sensitivity (90.0%) and specificity (88.9%) in blood samples using a cutoff value of MO1 > 94%. For BM samples, a reduced MO3 < 1.24% was more closely associated with CMML with a sensitivity of 96.0% and a specificity of 79.5%. A side-by-side comparison of our assay with the original “monocyte assay” showed strong agreement. Conclusions: Our study demonstrates the utility of a practical and robust approach for monocyte subset analysis in the diagnosis of CMML.

## Simple Summary

Flow cytometry monocyte partitioning has emerged as an important tool for the diagnosis of chronic myelomonocytic leukemia (CMML); however, its application in real-world settings remains limited. Here, we present the results of monocyte subset analysis using a routine 8-color myelomonocytic tube and a new gating strategy. We demonstrated that an increased classical monocyte fraction (cutoff, 94%) in the peripheral blood was able to distinguish CMML from non-CMML cases with high sensitivity (90.0%) and specificity (88.9%). For bone marrow samples, a decreased non-classical monocyte fraction (cutoff, 1.24%) was associated with CMML diagnosis, with a sensitivity of 96.0% and a specificity of 79.5%. Our study confirms the usefulness of monocyte partitioning in the diagnosis of CMML and extends previous studies by providing a practical approach that can be easily incorporated into daily clinical flow cytometry practice.

## 1. Introduction

Chronic myelomonocytic leukemia (CMML) is a myeloid neoplasm with features of both myelodysplastic syndrome (MDS) and myeloproliferative neoplasm (MPN). The diagnosis of CMML requires the presence of persistent peripheral blood monocytosis and morphologic dysplasia [1]. Approximately 30% of CMML patients have chromosome abnormalities and ~90% have somatic mutations, most commonly in the *TET2*, *SRSF2* and *ASXL1* genes [2,3,4]. However, monocytosis can be seen in many reactive and neoplastic conditions, and similar gene mutations are observed in other myeloid neoplasms and in clonal hematopoiesis of indeterminate potential (CHIP). Therefore, the diagnosis of CMML remains challenging and one of exclusion, especially in cases with minimal dysplasia and low monocytosis. Several groups have investigated immunophenotypic alterations in CMML using flow cytometry and found a spectrum of antigen aberrations, including the overexpression of CD56 and CD2 and decreased expressions of HLA-DR, CD13, CD11c and others [5,6,7,8,9,10]. These aberrancies are helpful in the diagnosis of myeloid neoplasms but are not entirely specific for CMML.

In recent years, monocyte subset partitioning using flow cytometry has emerged as a useful tool for the diagnosis of CMML. Human monocytes can be divided into three phenotypically and functionally distinct subpopulations based on CD14 and CD16 expression: the classical monocytes or MO1 (CD14++/CD16-), the intermediate monocytes or MO2 (CD14+/CD16+) and the non-classical monocytes or MO3 (CD14^dim^/CD16+) [11,12,13,14]. In healthy individuals, ~85% of the monocytes are MO1, ~5% are MO2 and ~10% are MO3 [13]. In their pioneering study, Selimoglu-Buet and colleagues demonstrated a characteristic increase in the classical monocyte fraction in CMML patients, compared to healthy donors and patients with reactive monocytosis or another hematologic malignancy [15]. A cutoff value of MO1 ≥ 94% was found to be associated with high specificity (95.1%) and sensitivity (90.6%). Several subsequent studies corroborated with this finding [16,17,18,19,20]. In the 2022 World Health Organization (WHO) classification, the abnormal partitioning of peripheral blood monocyte subsets using flow cytometry is included as one of the supporting diagnostic criteria for CMML [21].

Although the monocyte partitioning flow cytometry proposed by Selimoglu-Buet et al., now known as the “monocyte assay”, represents an important screening test for the diagnosis of CMML, its application in routine practice remains limited, partly due to the specific technical requirements. Several studies have evaluated the utility of monocyte partitioning assay in real-world settings with somewhat inconsistent results. Some showed low sensitivity ranging from 73% to 75% when using an MO1 cutoff of 94% [19,22,23], and one study failed to demonstrate a correlation between abnormal monocyte partitioning and CMML [24]. We sought to re-assess the usefulness of monocyte partitioning in the diagnosis of CMML by employing an 8-color antibody tube routinely used in our lab for lymphoma/leukemia phenotyping and our standard sample processing protocol. Here, we show that, by applying a new gating strategy, our assay effectively distinguishes CMML from non-CMML cases and can be easily incorporated into routine clinical practice.

## 2. Materials and Methods

### 2.1. Case Selection

The pathology database at Northwestern Memorial Hospital was retrospectively searched for CMML cases that had peripheral blood and/or bone marrow flow cytometry studies between September 2021 and July 2024. Cases with other myeloid neoplasms or non-neoplastic conditions that had flow cytometry immunophenotyping during the same period were collected as controls. The diagnosis of CMML and other myeloid neoplasms was based on the 2016 WHO criteria and was established using a combined evaluation of clinical/laboratory findings, morphologic and immunophenotypic features of peripheral blood and bone marrow samples, and cytogenetic and molecular results. This study was reviewed and approved by the Institutional Review Board of Northwestern University.

For peripheral blood analysis, a total of 56 cases were included, as follows: 20 with CMML, 15 with non-CMML myeloid neoplasms (non-CMML-MN) and 21 with no myeloid neoplasms (non-MN). The non-CMML-MN group consisted of 5 MDS, 6 MPN (3 primary myelofibrosis/PMF, 2 essential thrombocythemia/ET and one polycythemia vera/PV), 2 MDS/MPN NOS, 1 chronic myeloid leukemia (CML) and 1 acute myeloid leukemia (AML). The patients in the non-MN group had various reactive conditions including infection, lymphocytosis, solid tumors with leukopenia or leukocytosis, skin rash, splenomegaly, autoimmune disorders and others. For bone marrow studies, a total of 69 cases were included, as follows: 25 CMML, 28 non-CMML-MN and 16 non-MN. The non-CMML-MN group included 18 MDS patients, 8 MPN patients (4 PMF, 3 ET and 1 PV), 2 MDS/MPN NOS patients and 1 AML patient. The patients in the non-NM group had various conditions including lymphoma (negative staging marrow), multiple myeloma (in remission), mild thrombocytosis (reactive), leukopenia, iron deficiency anemia and others. None of the patients in the non-MN group had developed myeloid neoplasms during follow-up. A prospective study was performed to compare our monocyte partitioning method to the standard “monocyte assay”. This cohort included one CMML patient and 17 healthy individuals who had no history of malignancy.

### 2.2. Flow Cytometry

Peripheral blood and bone marrow samples were collected in EDTA and processed within 24 h. Following red blood cell lysis with ammonium chloride, the samples were incubated with antibodies at 4 °C for 15 min followed by one wash. Samples collected before April 2023 were analyzed with a Canto II cytometer (BD, Franklin Lakes, NJ, USA) using FACSDiva software (Version 8.0.2; BD Franklin Lakes, NJ, USA). After April 2023, samples were analyzed with a Lyric cytometer (BD, Franklin Lakes, NJ, USA) using Kaluza C software (Version1.1.; Beckman Coulter, Brea, CA, USA). The following antibody combination, as part of our routine lymphoma/leukemia immunophenotyping panel, was used for monocyte subset analysis: CD14 FITC (BD, clone MΦP9), CD64 PE (Beckman Coulter, clone 22), CD13 PE-Cy5.5 (Beckman Coulter, clone Immu103.44), CD34 PE-Cy7 (Beckman Coulter, clone 581), CD11b (BD, clone D12), CD16 APC-H7 (BD, clone 3G8), CD33 BV421 (BD, clone 398) and CD45 KrO (Beckman Coulter, clone J33). At least 50,000 events were acquired for each tube.

For comparison, we also used the standard “monocyte assay” published by Selimoglu-Buet et al. [16]. Briefly, the samples were incubated with antibodies at 4 °C for 15 min, followed by red cell lysis and immediate analysis (no wash). The following antibodies were used for monocyte analysis: CD14 FITC (BD, clone MΦP9), CD56 PE-Cy5.5 (N901), CD2 PE-Cy7 (Beckman Coulter, clone 39C1.5), CD24 APC (Biolegend, clone ML5), CD16 APC-H7 (BD, clone 3G8), CD45 KrO (Beckman Coulter, clone J33) and CD7 BV605 (BD, clone M-T701). At least 500,000 events were acquired for each tube.

### 2.3. Gating Strategy for Monocyte Subset Partitioning Using a Routine 8-Color Tube

**Figure 1:**
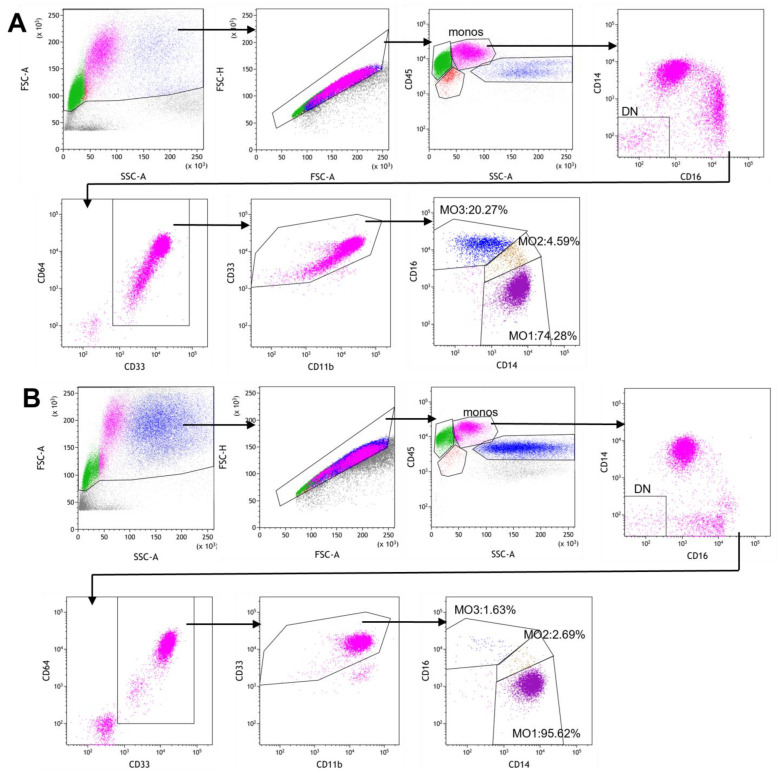
Gating strategy for monocyte subset analysis: After the selection of cellular events and singlets, monocytes were liberally gated as a CD45+/SSC intermediate population, which was then subjected to CD14 vs. CD16 gating to exclude CD14-/CD16- double negative events. The CD64+/CD33+ myelomonocytic population was then positively selected to exclude non-myeloid cells including the CD16+ NK cells. This population was further gated to exclude the residual granulocytes that were CD11b bright and CD33 intermediate. Finally, the “clean monocytes” were used for monocyte partitioning analysis to identify the classical monocyte (MO1, CD14++CD16-), intermediate monocyte (MO2, CD14+CD16+) and non-classical monocyte (MO3, CD14^dim^CD16+) subsets. (**A**) An example of peripheral blood analysis from a non-NM case with infection-associated reactive monocytosis. (**B**) An example of peripheral blood analysis from a CMML patient. Green: Lymphocytes; Pink: Monocytes; Dark Blue: Granulocytes; Purple: MO1; Brown: MO2; Blue: MO3.

In our routine screening lymphoma/leukemia flow cytometry immunophenotyping, an 8-color myelomonocytic tube (CD13/CD33/CD64/CD14/CD16/CD11b/CD34/CD45) was included to characterize granulocyte, monocyte and blast populations. Taking advantage of this existing tube, we designed a sequential gating strategy to obtain a “clean” monocyte population for subsequent partitioning analysis. As shown in Figure 1, after gating on cellular events and singlets, monocytes were liberally gated as the CD45+/SSC intermediate population, which was then subjected to CD14 vs. CD16 gating to exclude CD14-/CD16- double negative events that included residual T and B lymphocytes. The remaining events (including CD14+/CD16+, CD14+/CD16- and CD14-/CD16+ events) were subjected to CD64 vs. CD33 gating to positively select the CD64+/CD33+ myelomonocytic population and to exclude non-myeloid cells including the CD16+ NK cells. This population was further gated to exclude the residual granulocytes that were CD11b bright and CD33 intermediate. Finally, the “clean monocytes” were used for monocyte partitioning analysis to identify the classical monocyte (MO1, CD14++CD16-), intermediate monocyte (MO2, CD14+CD16+) and non-classical monocyte (MO3, CD14^dim^CD16+) subsets. Figure 1A illustrates a case of reactive monocytosis and Figure 1B demonstrates a confirmed CMML case with increased MO1 and decreased MO2 and MO3 fractions.

### 2.4. Statistical Analysis

Statistical analysis was performed using Prism 7 software (Version 1.05, GraphPad, San Diego, CA, USA) or Excel software (Version 2501, EXCEL, Baton Rouge, LA, USA). The Student’s *t*-test was used to compare groups. A receiver operating characteristic (ROC) curve analysis was performed to evaluate the relationship between sensitivity and specificity using a nonparametric approach. The cutoff value was estimated by maximizing the Youden Index (*J* = sensitivity + specificity-1) [25]. Linear regression was used for correlation analysis. A *p* value of ≤ 0.05 was defined as statistically significant.

## 3. Results

### 3.1. Monocyte Subset Analysis on Peripheral Blood Samples from CMML Patients and Non-CMML Controls

**Figure 2:**
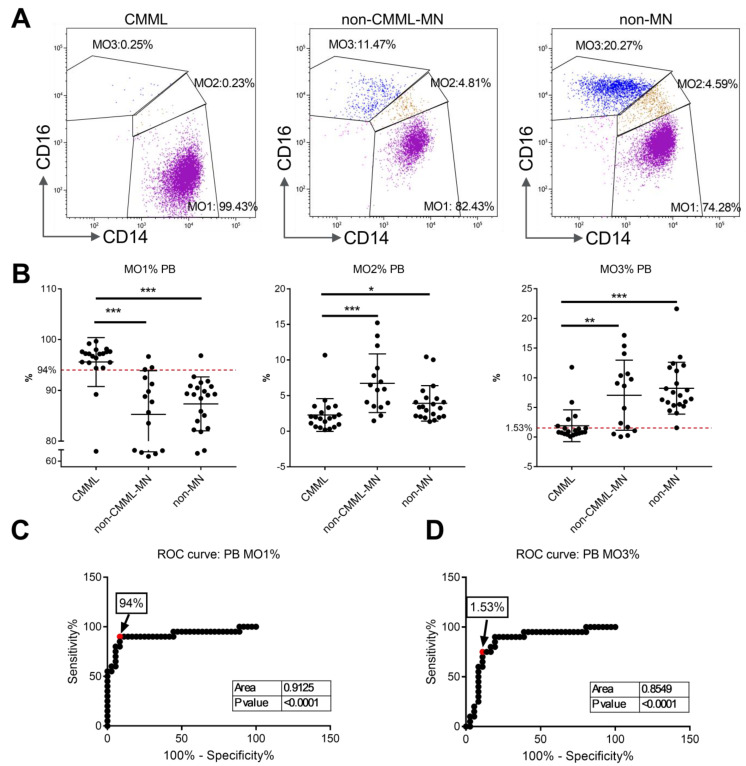
Percentages of monocyte subsets in peripheral blood samples from CMML and non-CMML patients: (**A**) representative flow cytometry dot plots from patients with CMML, with non-CMML myeloid neoplasms (non-CMML-MN) and with no history of myeloid neoplasm (non-MN). Purple: MO1; Brown: MO2; Blue: MO3. (**B**) Percentages of MO1, MO2 and MO3 monocyte subsets in patients with CMML, non-CMML-MN and non-MN. Red dashed lines indicate cut-off value of MO1 and MO3 based on ROC analysis. *: *p* < 0.05; **: *p* < 0.01; ***: *p* < 0.001. (**C**) Receiver operating characteristic (ROC) curve analysis of the peripheral blood MO1 percentages for CMML diagnosis. (**D**) Receiver operating characteristic (ROC) curve analysis of the peripheral blood MO3 percentages for CMML diagnosis.

The peripheral blood cohort included the following 56 cases: 20 with confirmed CMML, 15 with non-CMML myeloid neoplasms (non-CMML-MN) and 21 with no myeloid neoplasms (non-MN). Representative flow cytometry dot plots from patients with CMML, with non-CMML myeloid neoplasms (non-CMML-MN) and with no history of myeloid neoplasm (non-MN) are shown in Figure 2A. The monocyte subset analysis showed that there was a significantly higher accumulation of MO1 monocytes in CMML patients than in the non-CMML-MN and non-MN groups. As shown in Figure 2B, the mean MO1 percentage was 95.58% ± 4.8% for the CMML group, compared to 85.29% ± 8.6% for the non-CMML-MN group (*p* < 0.001) and 87.35% ± 5.3% for the non-MN group (*p* < 0.001). By applying a cutoff value of MO1 ≥ 94% for the diagnosis of CMML, the sensitivity was 90.0% and the overall specificity was 88.9% (80% for the non-CMML-MN group and 95.2% for non-MN group). A receiver operating characteristic (ROC) curve analysis was performed and revealed a similar cutoff value (94.9%) and an area under curve (AUC) of 0.9125 (Figure 2C).

MO2 percentages were lower in the CMML group (2.28% ± 2.3%) than in the non-CMML-MN group (6.74% ± 4.1%, *p* < 0.001) and the non-MN group (3.92% ± 2.5%, *p* = 0.035) (Figure 2B). Given the significant overlap between the CMML and non-MN groups, the diagnostic value of MO2 is deemed to be limited.

MO3 percentages were significantly lower in the CMML group (1.86% ± 2.7%) than in the non-CMML-MN (7.06% ± 5.9%, *p* = 0.001) and non-MN groups (8.23% ± 4.4%, *p* < 0.001) (Figure 2B). By applying the suggested cutoff value of MO3 < 1.13% for differentiating CMML from non-CMML [19], the sensitivity was low at 60.0%, and the overall specificity was 88.9% (73.3% for non-CMML-MN and 100% for non-MN). A recent study proposed a 2.5% cutoff value for MO3 [26]. Our ROC curve analysis revealed an optimal MO3 cutoff value of 1.53% and an AUC of 0.8507 (Figure 2D). By applying this value, the sensitivity was 75.0%, and the overall specificity was 88.9% (73.3% for the non-CMML-MN and 100% for non-MN). Therefore, a decreased MO3 fraction of ≤1.53% can be useful for the diagnosis of CMML, but the sensitivity is lower than the standard of MO1 ≥ 94%.

When applying a combination of MO1 > 94% and MO3 < 1.53%, the sensitivity was 75.0%, and the specificity was 94.4% (86.7% for the non-CMML-MN and 100% for non-MN). Therefore, a combination of increased MO1 > 94% and decreased MO3 < 1.53% produced a better specificity but suffered from a lower sensitivity than using MO1 > 94 alone.

### 3.2. Monocyte Subset Analysis on Bone Marrow Samples

**Figure 3:**
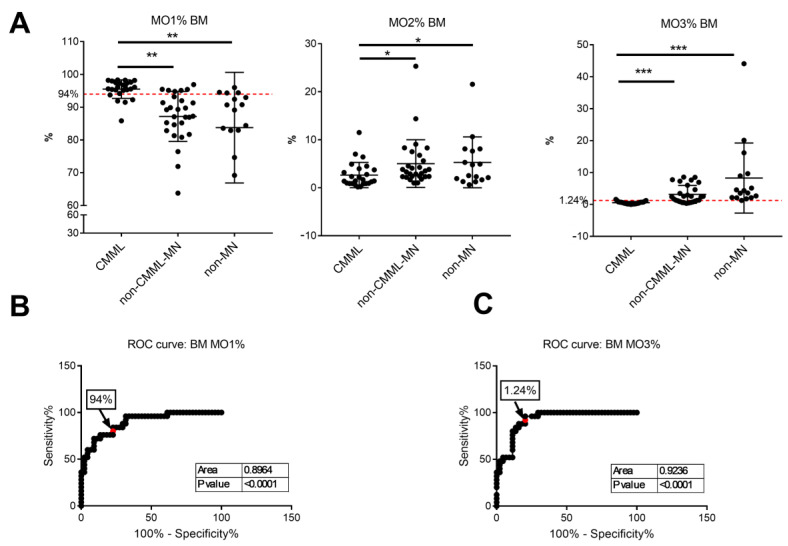
Percentages of monocyte subsets in bone marrow samples from CMML and non-CMML patients: (**A**) percentages of MO1, MO2 and MO3 monocyte subsets in patients with CMML, non-CMML-MN and non-MN. Red dashed lines indicate cut-off value of MO1 and MO3 based on ROC analysis. *: *p* < 0.05; **: *p* < 0.01; ***: *p* < 0.001. (**B**) Receiver operating characteristic (ROC) curve analysis of the bone marrow MO1 percentages for CMML diagnosis. (**C**) Receiver operating characteristic (ROC) curve analysis of the bone marrow MO3 percentages for CMML diagnosis.

To investigate whether monocyte partitioning can be applied to bone marrow for the diagnosis of CMML, bone marrow samples from 25 CMML patents, 28 non-CMML-MN patients and 16 non-MN patients were analyzed. Like peripheral blood, the percentages of MO1 in bone marrow samples were significantly higher in the CMML group (95.51% ± 2.8%) than in the non-CMML-MN (85.2% ± 7.6%, *p* < 0.001) and non-MN (83.8% ± 16.9%, *p* = 0.001) groups (Figure 3A). Using a cutoff value of MO1 > 94%, the sensitivity was 84%, and the overall specificity was 77.3% (78.6% for non-CMML-MN group and 75.0% for non-MN group). The ROC analysis revealed an optimal cutoff value of 93.5% and the AUC was 0.8964 (Figure 3B). Therefore, the previously reported 94% MO1 cutoff is useful for bone marrow analysis in the diagnosis of CMML, although the performance was not as good as the peripheral blood analysis.

The bone marrow MO2 percentages were slightly lower in the CMML group (2.64% ± 2.6%) than in the non-CMML-MN (5.02% ± 5.0%, *p* = 0.039) and non-MN (5.29% ± 5.3%, *p* = 0.037) groups (Figure 3A). Given the significant overlap between the three groups, the MO2 percentage alteration has limited diagnostic value.

The bone marrow MO3 percentages were significantly lower in the CMML group (0.54% + 0.4%) than in the non-CMML-MN (3.14% + 2.8%, *p* < 0.001) and non-MN (8.26% + 11.0%, *p* = 0.001) groups (Figure 3A). Using the previously suggested cutoff value of <1.13%, the sensitivity was 88%, and the overall specificity was 79.5% (67.9% for non-CMML-MN group and 100% for non-MN group). Our ROC curve analysis showed an optimal MO3 cutoff value of 1.24% and an AUC of 0.9236 (Figure 3C). By applying this cutoff value, the specificity was improved to 96.0%, and the overall specificity remained at 79.5% (67.9% for non-CMML-MN group and 100% for non-MN group). Therefore, for bone marrow samples, a decreased MO3 fraction (cutoff value, <1.24%) can differentiate CMML from non-MN cases with high sensitivity (96%) and specificity (100%). The specificity is lower when compared to the non-CMML-MN group, mainly because seven of the eighteen MDS cases in this group had MO3 < 1.24%. Some of these MDS cases developed overt monocytosis during follow-up and may represent an early stage of CMML (see discussion in Section 4).

### 3.3. Side-by-Side Comparison with the Standard “Monocyte Assay”

**Figure 4:**
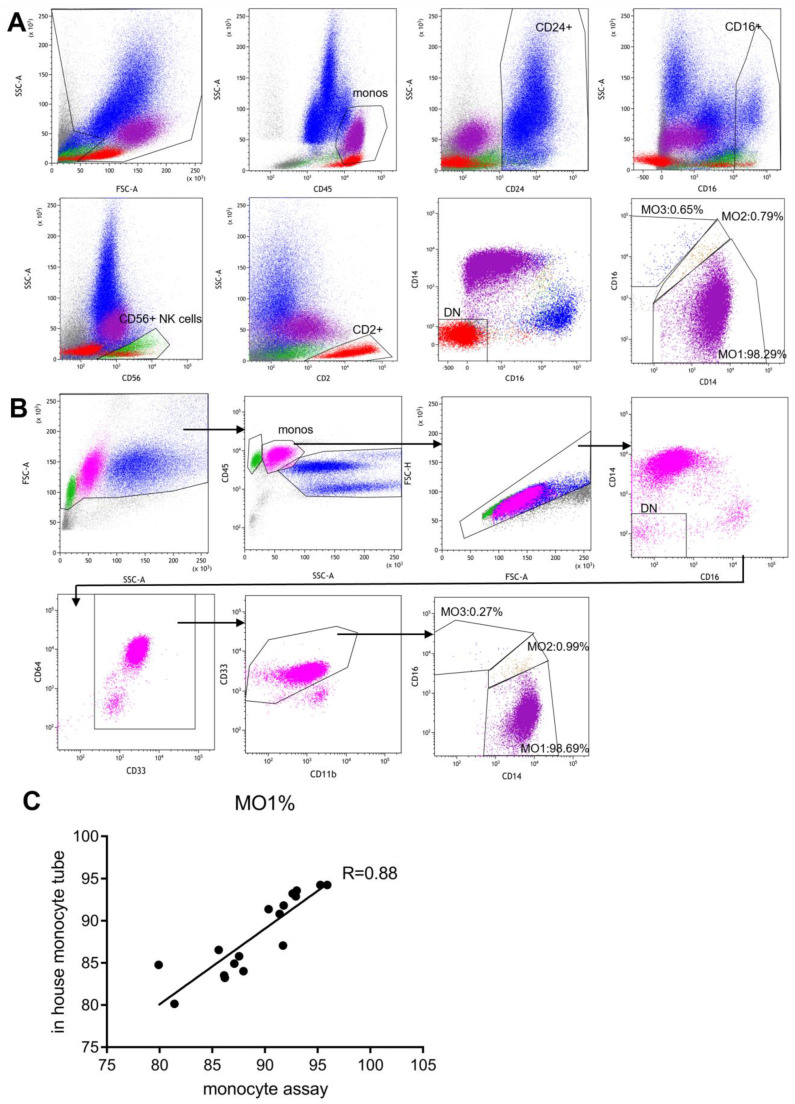
Side-by-side comparison of the published “Monocyte Assay” and our lab-developed monocyte assay. The figures show the gating strategies and results of a representative peripheral blood sample from a CMML patient by using (**A**) the published “Monocyte Assay” and (**B**) our own monocyte assay. (**A**) Blue: CD24+ B cells and granulocytes; Green: CD56+ SSC low NK lymphocytes; Red: CD2+ SSC low lymphocytes; Purple: MO1; Brown: MO2; Blue: MO3. (**B**) Green: Lymphocytes; Pink: Monocytes; Dark Blue: Granulocytes; Purple: MO1; Brown: MO2; Blue: MO3. (**C**) Linear regression analysis of the MO1 percentages of 18 peripheral blood samples between the two assays.

The original publication by Selimoglu-Buet et al. described a lyse-no-wash sample prep method and an exclusion gating strategy for monocyte subset repartitioning analysis [15]. This is now referred to as the “monocyte assay”. We prospectively analyzed 18 peripheral blood samples using both our routine method and the “monocyte assay”. The 18 samples included one CMML patient and 17 healthy individuals. A portion of the sample was processed according to the published “monocyte assay” protocol and stained with the recommended panel of six antibodies (CD14, CD16, CD2, CD24, CD56 and CD45). The data were analyzed according to the Selimoglu-Buet exclusion strategy and Boolean gating, as illustrated in Figure 4A. Another portion of the sample was processed and analyzed using our routine method (Figure 4B). The two methods showed similar monocyte partitioning results, and both successfully identified the CMML case with similar MO1 percentages (Figure 4A,B). The linear regression analysis showed a close agreement between the two methods (Figure 4C).

### 3.4. Clinical Features, Chromosome Abnormalities and Gene Mutations of CMML Patients

Our study included 38 CMML patients, of whom 6 had flow cytometric analysis on both peripheral blood and bone marrow samples, 14 had blood analysis only and 19 had bone marrow analysis only. There were 25 males and 13 females (male/female ratio = 1.9/1), with a median age of 72 years (range, 23–93 years). Regarding morphology, 28 patients had CMML-1 (blasts < 5% in blood and <10% in bone marrow) and 10 had CMML-2 (blasts 5–19% in blood or 10–19% in bone marrow). Based on White Blood Cell (WBC) count, there were 21 proliferative type CMML (pCMML) (WBC > 13 × 10^9^/L) and 17 dysplastic type CMML (dCMML) (WBC ≤ 13 × 10^9^/L). The CMML patients had a median hemoglobin level of 10.4 g/dL (range 6.4–14.9), a median absolute monocyte count (AMC) of 2.7 × 10^9^/L (range 1.0–29.5) and a median platelet count of 100 × 10^9^/L (range 11–313). Of the 36 patients with chromosome analysis, 22 had a normal karyotype and 14 had chromosome abnormalities (39%), with one showing a complex karyotype. Of the 31 patients with next generation sequencing studies, all but one had somatic mutations, most commonly *TET2* (16 cases), *SRSF2* (13 cases), *KRAS/NRAS* (10 cases), and *ASXL1* (7 cases). The mean number of mutations per patient was 2.9. The features of our CMML patients were comparable to the published data [27]. The clinical, laboratory and molecular/genetic data of the CMML patients are summarized in Table 1.

For the six CMML patients who had both peripheral blood and bone marrow flow cytometry studies, monocyte subset distributions were comparable between blood and bone marrow samples. Both peripheral blood and bone marrow showed an increased MO1 fraction > 94% and a decreased MO3 fraction < 1.24% in all six patients. By analyzing monocyte subset distributions in CMML patients with various clinical and genetic characteristics, we found no correlation between MO1 or MO3 percentages and the subtypes of CMML (CMML-1 vs. CMML-2 or pCMML vs. dCMML), the presence or absence of chromosome abnormalities or the number and type of gene mutations.

## 4. Discussion

The standard “monocyte assay”, as described by Selimoglu-Buet et al., is an important tool for the screening and diagnosis of CMML [15,16,17,18,20], but it has some technical constraints. The assay requires a specific lyse-no-wash procedure, a minimum of 50,000 events in the monocyte gate, a specific panel of antibodies, and an exclusion and Boolean gating strategy. These are not routinely used by most of the clinical flow cytometry laboratories. We have routinely used an 8-color myelomonocytic tube as part of our screening panel for lymphoma/leukemia immunophenotyping. This tube (CD14, CD16, CD13, CD33, CD64, CD11b, CD34, CD45) was designed to characterize granulocyte, monocyte and blast populations. It is particularly useful in evaluating abnormal CD11b/CD13/CD16 maturation patterns in granulocytes and in distinguishing dysplastic/hypogranular granulocytes from blasts and monocytes. It included several markers to positively select and characterize monocytes. Taking advantage of this existing panel, we retrospectively analyzed the three monocyte subsets on 56 peripheral blood samples using a new sequential gating strategy. By applying a cutoff value of MO1 ≥ 94%, the assay was able to distinguish CMML from non-CMML cases with high sensitivity (90.0%) and specificity (88.9%). The results are comparable to those reported previously using the standard “monocyte assay” [15,16,18,26]. Our gating strategy is straightforward (Figure 1) and can be easily adapted by regular clinical flow cytometry laboratories.

To further validate our method, we conducted a side-by-side comparison with the published “monocyte assay” on 18 peripheral blood samples. The results demonstrated a strong correlation between the two methods. During this validation process, we realized that the “monocyte assay” requires analysis by a highly skilled flow cytometry expert, and it constitutes a separate assay rather than an integrated part of routine leukemia/lymphoma immunophenotyping. These constraints made it difficult to implement the “monocyte assay” in our very busy clinical flow cytometry lab. The routine myelomonocyte assay, when combined with a new gating strategy, can be easily incorporated into daily practice to provide useful information in the differential diagnosis of CMML.

One limitation of our assay is the lack of T and NK cell markers in the 8-color tube. But a two-step gating strategy, i.e., the exclusion of the CD14-/CD16- population and the positive selection of the CD33+/CD64+ population, effectively eliminates the small number of lymphocytes remaining in the initial monocyte gate, as B and T cells will fall into the DN gate and NK cells and any residual non-myeloid cells/debris will not be included in the CD33+/CD64+ gate. A recent study by Jurado et al. [28] compared different gating strategies in monocyte partitioning analysis, and suggested the use of monocyte positive gating to exclude NK cells. In addition, neoplastic monocytes in CMML can express CD56 and, to a lesser extent, CD2. Therefore, gating by exclusion requires great caution to not exclude abnormal monocytes. We recognize that CD64 expression is dimmer on non-classical monocytes [12], and therefore our gating on CD64 is generous (Figure 1). A comparison with the standard “monocyte assay” showed comparable “clean” monocyte population, further validating our assay. In the future, we plan to add CD2 and CD56 to this tube as we switch to a 10-color screening panel.

Despite the good performance of the monocyte partitioning flow cytometry assay in the diagnosis of CMML, there are several false negative and false positive cases. In the peripheral blood cohort, two of the 20 CMML cases had MO1 < 94%. One was a 23-year-old young man with a very low MO1 percentage (77%). He had *PTPN11* and *BCOR* mutations and significant hepatosplenomegaly, suggestive of juvenile myelomonocytic leukemia (JMML) features. The patient had an aggressive clinical course and died within 20 months of diagnosis despite multiple lines of therapy and stem cell transplant. It has been reported that pediatric JMML patients have previously presented with decreased MO1 fractions, as seen in our patient [29,30]. Therefore, CMML cases with a particularly low MO1 fraction and JMML-type mutations may represent a distinct type of disease different from typical adult CMML. Further studies are needed to examine this notion. The other false negative CMML case in the peripheral blood cohort with a low MO1 (89%) was a 71-year-old female with CMML-1 and KRAS mutation. She had no inflammatory disorders and had not received any therapy. It is unclear why this case had low MO1. We also found several false positive cases. Four of 36 non-CMML cases in the peripheral blood cohort had MO1 > 94%: one had lung cancer with leukocytosis, two had MPN and one had MDS/MPN NOS. These observations emphasize the importance of a comprehensive approach to CMML diagnosis.

Most previous monocyte partitioning studies focused on peripheral blood analysis. At our institution, however, most of the patients with clinical suspicion for myeloid neoplasms usually undergo a bone marrow biopsy without peripheral blood flow cytometry studies. We therefore sought to investigate monocyte subset partitioning in bone marrow samples and to ask whether this information can be helpful in differentiating between CMML and non-CMML. Prior studies on bone marrow monocyte partitioning are limited, and the results are not always consistent [19,26,31]. One earlier study on a small cohort of patients showed a sensitivity of 60% and a specificity of 92% using a cutoff of MO1 > 94%, but the sensitivity improved when using a decreased MO3 fraction [19]. A recent study using the “monocyte assay” demonstrated 87% sensitivity and 80% specificity using a cutoff of MO1 > 94 [26]. We analyzed 69 bone marrow samples (25 CMML and 44 non-CMML cases) and revealed a satisfactory sensitivity of 84.0% and specificity of 77.3% when using a cutoff of MO1 > 94%. Interestingly, a decrease in MO3 fraction showed better association with CMML in bone marrow samples, similar to the previous study by Hudson at al [19]. When we applied an MO3 cutoff value of 1.24% (optimal cutoff in our bone marrow cohort), the sensitivity was 96.0% and the overall specificity was 79.5%. These findings support the notion that bone marrow monocyte subset partitioning by flow cytometry can be a helpful tool in the diagnosis of CMML, and a decreased MO3 fraction is of particular value in bone marrow evaluation.

In our cohort, there were seven MDS cases with a relative “monocytosis” (monocytes > 10% of WBC, but absolute monocyte count <0.6 × 10^9^/L). Interestingly, four of the seven cases exhibited increased MO1 > 94% and MO3 < 1.24% in bone marrow flow cytometric analysis. Three of the four patients had developed more overt monocytosis within a few months (>17%, >1.0 × 10^9^/L). On the other hand, none of the three patients with MO1 < 94% developed monocytosis during follow-up; in fact, their monocyte percentages decreased to <10%. These findings corroborate with previous reports that a subset of MDS patients had increased MO1 above 94% [16,20], and approximately half of these patients had evolved to overt CMML [16,32]. It has been proposed that these CMML-like MDS cases may represent an early stage of the CMML continuum [33,34]. In recognizing this phenomenon, the current WHO and ICC classification reduced the absolute monocyte count requirement from 1.0 × 10^9^/L to 0.5 × 10^9^/L for the diagnosis of CMML. It is not clear whether these CMML-like MDS constitute a different disease from the typical CMML or merely an early stage of CMML. Previous studies on the mutational profile of CMML-like MDS have shown that these cases tend to carry *TET2* mutations, often biallelic *TET2* mutations [32]. Three of our four CMML-like MDS patients had NGS data and none had *TET2* mutations, while two had *SRSF2* mutations. Further studies of MDS cases with abnormal distributions of monocyte subsets are needed to better understand their biological and genetic characteristics and potentially help to design better treatment.

## 5. Conclusions

Our study confirms that peripheral blood monocyte partitioning by flow cytometry is a powerful tool for CMML diagnosis. By combining a new and straightforward gating strategy with a common 8-color myelomonocytic antibody panel and routine lab protocol, we further demonstrate the practical utility of this analysis in a real-world setting and provide useful information to help bring this assay to daily clinical practice. Moreover, our analysis of bone marrow samples showed a strong association of CMML with a decreased percentage of non-classical monocytes, indicating its usefulness as a diagnostic marker for CMML in bone marrow evaluation.

## Figures and Tables

**Table 1:** Clinical, molecular/genetic and laboratory characteristics of patients with chronic myelomonocytic leukemia (CMML).

Age Median (Range)		72 (23–93)
Sex	Male	25
	Female	13
WBC (10^9^/L), median (range)		11.3 (2.7–84.7)
AMC (10^9^/L), median (range)		2.5 (1.0–24.6)
Monocyte %, median (range)		23% (9–44%)
Hemoglobin (g/dL), median (range)		10.4 (6.4–14.9)
Platelets (10^9^/L), median (range)		100 (11–313)
Cytogenetics (n = 36)	Normal karyotype	22
	Abnormal (non-complex)	13
	Abnormal (complex)	1
Next-generation sequencing (n = 31)		
Epigenetic regulator	*TET2*	16
	*ASXL1*	7
	*E2H2*	4
	*BCOR*	4
Spliceosome components	*SRSF2*	13
	*SF3B1*	2
	*U2AF1*	4
Transcription factor	*RUNX1*	6
Signal genes	*KRAS/NRAS*	10
	*CBL*	3
	*JAK2*	0
	*SH2B3*	4
	*PTPN11*	2
Others	*TP53*	1
	*SETBP1*	2
	*PHF6*	2
CMML subtypes		
	CMML-1	28
	CMML-2	10
	CMML-P	21
	CMML-D	17

## Data Availability

Data are contained within the article.
